# Branch-Chain-Rich Diisopropyl Ether with Steric Hindrance Facilitates Stable Cycling of Lithium Batteries at − 20 °C

**DOI:** 10.1007/s40820-024-01419-z

**Published:** 2024-05-16

**Authors:** Houzhen Li, Yongchao Kang, Wangran Wei, Chuncheng Yan, Xinrui Ma, Hao Chen, Yuanhua Sang, Hong Liu, Shuhua Wang

**Affiliations:** State Key Laboratory of Crystal Materials, Shandong University, Jinan, 250100 People’s Republic of China

**Keywords:** Solvation structure, Li metal battery, Low temperature, Steric hindrance, Disorder

## Abstract

Li metal batteries (LMBs) offer significant potential as high energy density alternatives; nevertheless, their performance is hindered by the slow desolvation process of electrolytes, particularly at low temperatures (LT), leading to low coulombic efficiency and limited cycle stability. Thus, it is essential to optimize the solvation structure thereby achieving a rapid desolvation process in LMBs at LT. Herein, we introduce branch chain-rich diisopropyl ether (DIPE) into a 2.5 M Li bis(fluorosulfonyl)imide dipropyl ether (DPE) electrolyte as a co-solvent for high-performance LMBs at − 20 °C. The incorporation of DIPE not only enhances the disorder within the electrolyte, but also induces a steric hindrance effect form DIPE’s branch chain, excluding other solvent molecules from Li^+^ solvation sheath. Both of these factors contribute to the weak interactions between Li^+^ and solvent molecules, effectively reducing the desolvation energy of the electrolyte. Consequently, Li (50 μm)||LFP (mass loading ~ 10 mg cm^−2^) cells in DPE/DIPE based electrolyte demonstrate stable performance over 650 cycles at − 20 °C, delivering 87.2 mAh g^−1^, and over 255 cycles at 25 °C with 124.8 mAh g^−1^. DIPE broadens the electrolyte design from molecular structure considerations, offering a promising avenue for highly stable LMBs at LT.

**Supplementary Information:**

The online version contains supplementary material available at 10.1007/s40820-024-01419-z.

## Highlights

- Branch chain-rich diisopropyl ether (DIPE) was selected as co-solvent of low-temperature electrolyte for lithium metal battery.
- The introduction of DIPE improved the disorder of electrolyte and the branch chains from DIPE exclude other solvents from the Li^+^ solvent sheath, thereby achieving a rapid desolvation process.
- The electrolyte guaranteed a uniform Li stripping and deposition during cycling at both room temperature and low temperature and ensured stable cycling performance for Li||LFP cells over 650 cycles at − 20 °C.

**Supplementary Information:**

The online version contains supplementary material available at 10.1007/s40820-024-01419-z.

## Graphical abstract

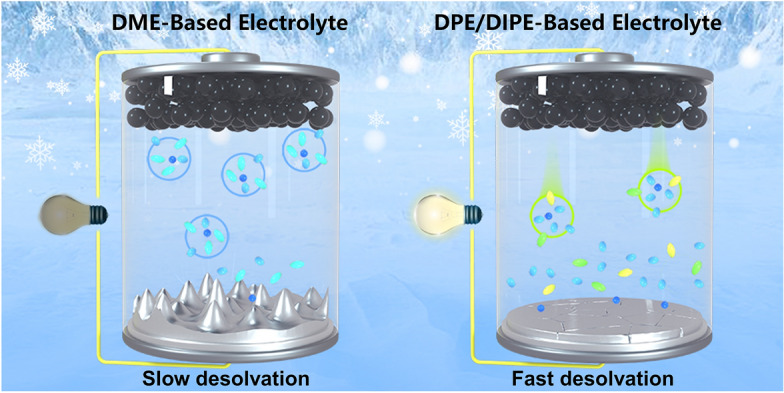

## Introduction

Li-ion batteries (LIBs) have been employed successfully in various applications for many years. However, the increasing demand for smart portable devices and long-range electric vehicles has revealed the limitations of LIBs due to the relatively low energy density resulting from the use of graphite anodes, which have a specific capacity of only 372 mAh g^−1^ [1–3]. In contrast, Li metal batteries (LMBs) hold great promise as the next generation of high energy density batteries, with Li offering an exceptionally high theoretical specific capacity of 3,860 mAh g^−1^ and a low redox potential (–3.04 V vs. the standard hydrogen electrode) [4–6]. Despite their potential, LMBs face significant challenges, particularly in low-temperature (LT) environments. These challenges include uncontrolled dendrite growth, the formation of “dead Li”, and continuous side reactions between Li and the electrolyte. These issues result in reduced energy density, low coulombic efficiency (CE), and short cyclability of LMBs [7, 8]. The poor performance of LMBs at LT [9, 10] can be attributed to increased charge transfer resistance (*R*_ct_) at the solid electrolyte interphase (SEI) and sluggish ion diffusion within the bulk electrolyte. Notably, the desolvation of the electrolyte at LT plays a significant role in these challenges [11, 12]. Generally, the commercialized electrolytes, such as ethylene carbonate (EC)-based electrolyte and dimethoxy ethane (DME)-based electrolyte, will generate a strong affinity between Li^+^ and solvents, thereby causing a sluggish desolvation kinetics and an inferior electrochemical performance in LMBs at LT [13–17].

Researchers have paid their attention on developing novel electrolytes with modified solvation structure, thereby improving the desolvation kinetics and electrochemical stability of LMBs at LT [18–21]. High-concentration electrolytes (HCEs), such as 10 M Li bis(fluorosulfonyl)imide (LiFSI) in dimethyl carbonate (DMC) [22], and 4.6 M LiFSI mixed 2.3 M LiTFSI in DME [23], have been proposed. By this design, more anions such as FSI^–^ in the solvation sheath of lithium in the electrolyte were increased, which promotes the formation of a stable SEI and rapid interfacial reaction kinetics [19, 20, 24]. Additionally, the introduction of low-viscosity diluents, such as 1,1,2,2-tetrafluoroethyl-2,2,3,3-tetrafluoropropyl ether [25] and bis(2,2,2-trifluoroethyl) ether [26], into HCEs has been explored to create locally HCEs. These diluents modify the solvent shell and enhance battery stability at LTs. Similarly, selecting solvents with low polarity, such as adding CO_2_ to low-polarity fluoromethane to create a liquified gas electrolyte, has been shown to yield an electrolyte with weak solvating characteristics [24, 27], ensuring low viscosity and excellent battery performance at LTs. Furthermore, fluorinated electrolytes, such as 2,2-dimethoxy-4-(trifluoromethyl)-1,3-dioxolane [28], can reduce the electron-donating ability of solvent oxygen atoms, resulting in weaker coordination with Li. In addition, locally concentrated ionic liquid electrolyte (LCILE) [18] is also a promising electrolyte for LMBs at LT. For example, LCILE composed by LiFSI, the 1-ethyl-3-methylimidazolium cation, bis(fluorosulfonyl)imide anion and 1,2-difluorobenzene [29] was also demonstrated favorable use for LMBs at − 20 °C*.* Recent studies have also explored the use of single oxygen donor ethers, such as diethyl ether [1] or dibutyl ether (CH_3_(CH_2_)_3_O(CH_2_)_3_CH_3_) [15], as promising electrolytes for Li–S systems in LT environments. Despite these advancements, problems, such as low CE and limited battery cycle life, still hinder the practical application of LMBs at LTs. Therefore, optimizing the solvent structure of the electrolyte to achieve rapid desolvation and enhance electrochemical performance in LT conditions remains a crucial challenge.

In this study, we introduced branch chain-rich diisopropyl ether (DIPE) and its isomer, dipropyl ether (DPE), as components of the solvent. 2.5 M LiFSI was dissolved in this solvent to create a weak solvating electrolyte for LMBs operating at − 20 °C. The presence of branch-rich DIPE enhanced the reversibility of Li anodes at both room temperature (RT, 25 °C) and LT conditions. Compared to electrolytes based on DME or DPE alone, the DPE/DIPE system reduced the number of solvent molecules and increased the participation of FSI^–^ ions in the Li^+^ first solvent shell, as confirmed by theoretical calculations. The enhanced diversity in the solvation structure and the resulting disorder in the 2.5 M LiFSI DPE/DIPE system are likely to weaken the binding of Li^+^ ions with the solvents. Besides, electrostatic potential (ESP) distribution analysis demonstrated that single oxygen ligand DPE and DIPE exhibit weak binding affinity with Li^+^, further reducing the interaction between Li^+^ and solvents in the solvent sheath. Noncovalent interactions (NCIs) revealed that the steric hindrance effect arising from DIPE’s branch chains repels DPE from the primary solvent sheath of Li^+^. Consequently, the weak binding ability of the single oxygen donor ethers and the steric hindrance from DIPE reduce the desolvation energy barrier of the electrolyte. In summary, optimizing the solvent structure resulted in a fast desolvation process of the electrolyte, leading to uniform Li stripping and deposition during cycling at both RT and LTs. Notably, the Li||Cu cell achieved an impressive CE of 98.70% at − 20 °C, and the designed electrolyte ensures stable cycling performance for Li||LFP (with a mass loading of approximately 10 mg cm^−2^) cell over 650 cycles, delivering a capacity of 87.2 mAh g^−1^ at 0.1 C and a temperature of − 20 °C. This study highlights the significance of solvent structure optimization in enhancing the electrochemical performance of LMBs, particularly in LT environments.

## Experimental Section

### Preparation of Electrolytes

Prior to use, DME, DPE, and DIPE were dried for 24 h. LiFSI was then added to DME, DPE, and differernt mixtures of DPE/DIPE to create 2.5 M electrolytes.

### Preparation of the Cathode

LiFePO_4_ cathode with an active material content of 91.5% was prepared by mixing LiFePO_4_, carbon black, and polyvinylidene fluoride powder in N-methyl-2-pyrrolidone solvent. An Al foil was evenly coated with the slurry and dried for 12 h at 60 °C, then 4 h at 85 °C to remove moisture.

### Characterization

Before characterization, each sample was cleaned three times with DME. SEM (S-4800) was employed to examine morphologies. XPS was performed using AXIS SUPRA with an Al Kα X-ray source (1,486.71 eV photons). ^7^Li NMR spectra were acquired using a Bruker 600 MHz spectrometer, calibrated with 1 M LiCl D_2_O solution as an external reference. Raman spectra were obtained using a Horiba LabRAM HR Evolution with a 633 nm laser source.

### Electrochemical Methods

Li||Li coin cells were assembled using two 150 μm Li foils. Li||Cu half cells consisted of 150 μm Li foils and Cu foils, to remove the impurities on the copper and activated the electrodes, the voltage was set at 0–1 V, and the current density was set at 0.1 mA cm^−2^. Li||LFP full cells were created by combining a prepared LiFePO_4_ cathode with a 50 μm Li foil in an ether-based electrolyte, with 70 μL the electrolyte added to the coin cells. Electrochemical experiments were conducted using a LAND system (CT2001A). Electrochemical impedance spectroscopy measurements were carried out using a CHI 600E electrochemical workstation on Li||Li-ion cells at various temperatures, with frequencies ranging from 10 to 100 kHz.

### Computational Methods

Classical MD simulations were conducted to investigate mixed solutions at the atomic level. Three bulk cases (System 1, System 2, and System 3) were created for MD simulations. System 1 included 3,000 DME and 1,161 LiFSI molecules; System 2 consisted of 1,122 LiFSI and 3,000 DPE molecules; and System 3 comprised 2,143 LiFSI, 3,000 DPE, and 3,000 DIPE molecules. The initial configurations of these systems were generated using the PACKMOL software [30], with molecules randomly placed in cubic simulation boxes. The OPLSAA force field [31, 32] was utilized to describe the molecules, encompassing both bonded and nonbonded interactions. Equations 1 and 2 represented the van der Waals (vdW) and electrostatic interactions, respectively, as integral components of the nonbonded interaction.

1 $$E_{LJ} \left( {r_{ij} } \right) = 4\varepsilon_{ij} \left( {\left( {\frac{{{\upsigma }_{ij} }}{{r_{ij} }}} \right)^{12} - \left( {\frac{{{\upsigma }_{ij} }}{{r_{ij} }}} \right)^{6} } \right)$$

2 $$E_{c} (r_{ij} ) = \frac{{q_{i} q_{j} }}{{4\pi \varepsilon_{o} \varepsilon_{r} r_{ij} }}$$

Equation 3 employs the Lorentz–Berthelot mixed rules for vdW interactions involving different types of atoms. Long-range electrostatic interactions were computed using the particle mesh Ewald method, with a cut-off distance of 1.2 nm for both vdW and electronic interactions.

3 $$\sigma_{ij} = \frac{1}{2}\left( {\sigma_{ii} + \sigma_{jj} } \right);\;\varepsilon_{ij} = \left( {\varepsilon_{ii} *\varepsilon_{jj} } \right)^{{{1 \mathord{\left/ {\vphantom {1 2}} \right. \kern-0pt} 2}}}$$

Energy minimization was initially employed to relax the simulation box. Subsequently, the simulation box was optimized within an isothermal-isobaric (NPT) ensemble using a time step of 1.0 fs, with temperature and pressure settings of 300 K and 1.0 atm, respectively. Temperature control was achieved using a Nose–Hoover thermostat, and pressure was maintained using a Parrinello-Rahman barostat. An NPT simulation time of 50.0 ns was selected as it proved sufficient for stabilizing the box size. The optimal configuration of the simulated box will be displayed in the following discussion. The velocity Verlet approach was employed to solve classical Newton’s equations, governing atomic motion throughout the MD simulation. All MD simulations were conducted using the GROMACS 2021.5 package [33].

**Fig. 1:**
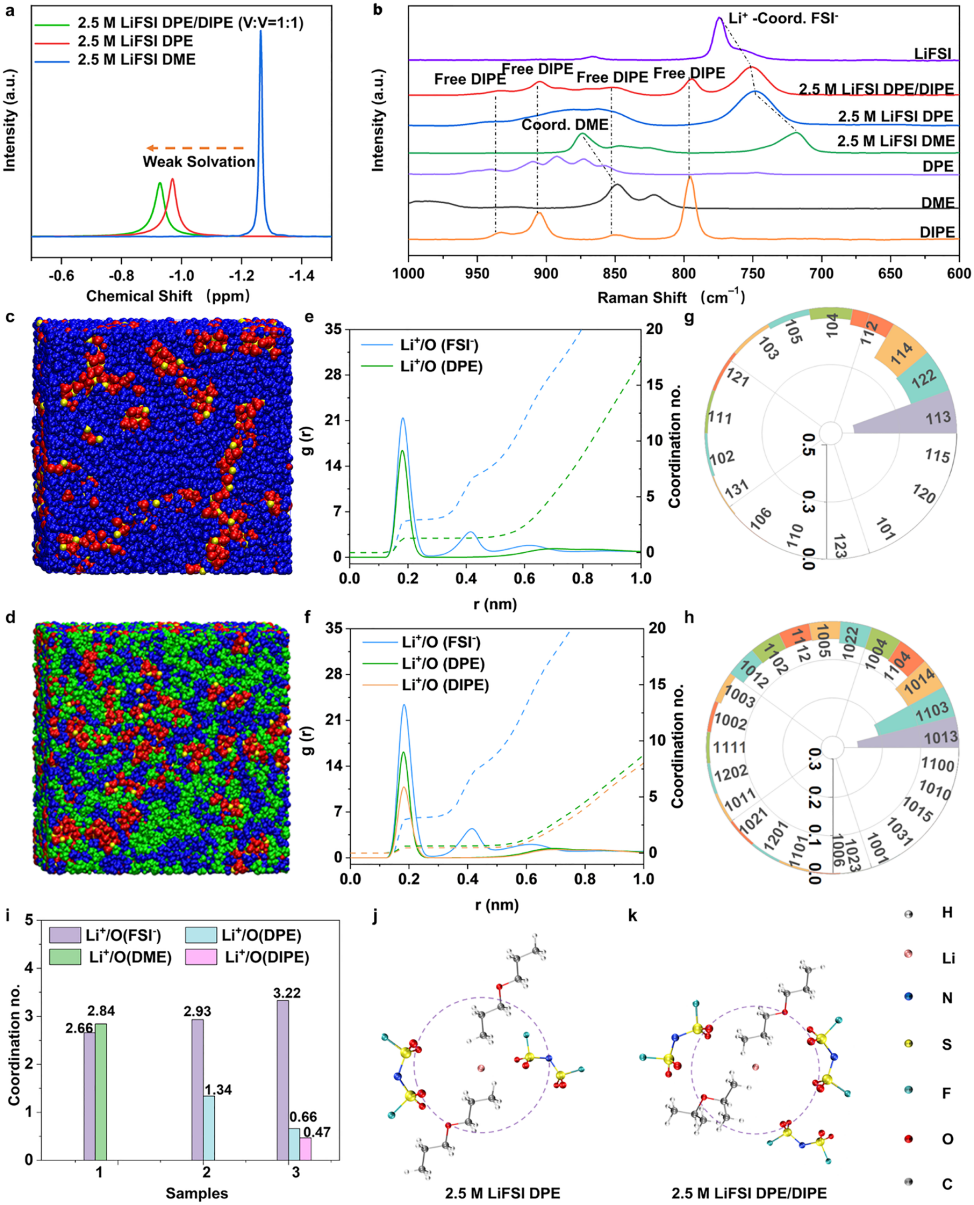
**a** ^7^Li NMR spectra of the three electrolytes. **b** Raman spectrum of the three electrolytes and corresponding components. Snapshot (yellow parts are Li^+^, red parts are FSI^–^, blue parts are DPE, green for DIPE) for **c** 2.5 M LiFSI DPE and **d** 2.5 M LiFSI DPE/DIPE. **e** Li^+^ radial distribution function (RDF) derived from MD simulations in 2.5 M LiFSI DPE. **f** Li^+^ radial distribution function (RDF) derived from MD simulations in 2.5 M LiFSI DPE/DIPE. Solvent structures for **g** 2.5 M LiFSI DPE and** h** 2.5 M LiFSI DPE/DIPE (Detailed information can be found in Tables S3 and S4). **i** Average coordination numbers in the first solvent shell of the three electrolytes ((1) 2.5 M LiFSI DME, (2) 2.5 M LiFSI DPE, and (3) 2.5 M LiFSI DPE/DIPE). Schematic solvent shell structures of **j** 2.5 M LiFSI DPE and **k** 2.5 M LiFSI DPE/DIPE

Grid data for NCI [34–40] analysis was generated using Multiwfn 3.730 and subsequently visualized using VMD.

## Results and Discussion

To address the challenges associated with LT operation of LMBs, we explored the use of DPE, characterized by a low melting point (− 123 °C) and single oxygen donor properties, as a solvent for LMB electrolytes. Specifically, we dissolved 2.5 M LiFSI in DPE (the molar ratio of LiFSI to DPE is ~ 250:998), which was denoted as 2.5 M LiFSI DPE. DME is a common solvent in ether-based electrolytes. For comparison, we prepared 2.5 M LiFSI in DME (the molar ratio of LiFSI to DME is ~ 250:770), designated as 2.5 M LiFSI DME. To further optimize the solvent structure of Li^+^, we introduced branch chain-rich DIPE as a co-solvent into 2.5 M LiFSI DPE, creating 2.5 M LiFSI DPE/DIPE with a volume ratio of V_DPE_:V_DIPE_ set at 1:1, 5:3 and 3:5 (the molar ratio of LiFSI to DPE to DIPE is ~ 250:499:506, 250:453:266 and 250:272:444, respectively). To confirm the superiority of DPE/DIPE system, 2.5 M LiFSI electrolytes with DME/DIPE (V_DME_:V_DIPE_ = 1:1) and DIPE alone were also prepared, denoted as 2.5 M LiFSI DME/DIPE and 2.5 M LiFSI DIPE, respectively.

Different characterizations of all the electrolytes abovementioned are discussed in Table S1. The 2.5 M LiFSI DME, 2.5 M LiFSI DPE, 2.5 M LiFSI DPE/DIPE (V_DPE_:V_DIPE_ set at 1:1 and 5:3) can completely dissolve the lithium salts. Unfortunately, 2.5 M LiFSI DPE/DIPE with V_DPE_:V_DIPE_ = 3:5 did not completely dissolve LiFSI. Moreover, it was observed that upon complete dissolution of the lithium salt, the 2.5 M LiFSI DME/DIPE and 2.5 M LiFSI DIPE demonstrated significant stratification (digital picture was in Fig. S1). Ionic conductivity was also performed, 2.5 M LiFSI DME electrolyte exhibits a significantly high ionic conductivity at 20 °C, approximately 7.502 mS cm^−1^, while the ionic conductivities of 2.5 M LiFSI DPE, 2.5 M LiFSI DPE/DIPE (V_DPE_:V_DIPE_ = 5:3), and 2.5 M LiFSI DPE/DIPE (V_DPE_:V_DIPE_ = 1:1) are ~ 2.077, 2.027, and 2.003 mS cm^−1^, respectively. Furthermore, we evaluated the CE at RT in different electrolytes using Li||Cu cells. Each cell was tested at 1 mA cm^−2^ and 1 mAh cm^−2^ (as depicted in Fig. S2). The cell in 2.5 M LiFSI DME exhibited poor cyclability, with only 111 cycles and an average CE of ~ 98.29%. In contrast, the cell in 2.5 M LiFSI DPE displayed over 334 cycles with an average CE value of ~ 98.98%. The cell in 2.5 M LiFSI DPE/DIPE (V_DPE_:V_DIPE_ = 5:3) endured over 300 cycles with an average CE of ~ 98.95%. Remarkably, the cell in 2.5 M LiFSI DPE/DIPE (V_DPE_:V_DIPE_ = 1:1) electrolytes demonstrated a CE of ~ 99.06% and the longest life over 390 cycles (Fig. S2). Considering all the above performances, we used 2.5 M LiFSI DPE/DIPE as the electrolyte for subsequent cell performance evaluation, in which the volume ratio of V_DIPE_:V_DPE_ was 1:1.

Effective wetting of the separator by the electrolyte is crucial for facilitating Li^+^ transmission [41]. Contact angles of the three electrolytes on the Celgard 2325 separator are shown in Fig. S3a–c. Notably, the 2.5 M LiFSI DME exhibited poor wettability, with a contact angle of 70.5°. In contrast, the contact angle was reduced to 56.9° for 2.5 M LiFSI DPE. Moreover, the 2.5 M LiFSI DPE/DIPE electrolyte showed a further decrease in the contact angle to 42.7°, indicating superior wettability and faster Li^+^ transmission.

To assess the solvent structures of these electrolytes, we conducted nuclear magnetic resonance (NMR) spectroscopy, which revealed distinct Li^+^ peaks in ^7^Li NMR spectra (Fig. 1a). Specifically, the peaks for 2.5 M LiFSI DME, 2.5 M LiFSI DPE, and 2.5 M LiFSI DPE/DIPE were approximately at − 1.26, − 0.97, and − 0.93 ppm, respectively. The shift toward more positive values (downfield) from 2.5 M LiFSI DME to 2.5 M LiFSI DPE, and further to 2.5 M LiFSI DPE/DIPE, is attributed to the weak complexation ability of oxygen in DPE/DIPE. This weak coordination results in a lower electron density around Li^+^ in 2.5 M LiFSI DPE/DIPE. Raman spectrum was also utilized to clarify the Li^+^ solvent shell. It has been reported that the peaks corresponding to the FSI^–^ of the LiFSI salt go through a notable red shift upon dissolution because of the decreased coordination between the Li^+^ with FSI^–^ and the enhanced coordination between the Li^+^ and solvents [1]. As shown in Fig. 1b, the S–N–S bending vibrations of FSI^–^ in the solid LiFSI salt were observed at 774 cm^−1^. The corresponding peaks in 2.5 M LiFSI DME shifted to 719 cm^−1^, while those in 2.5 M LiFSI DPE and 2.5 M LiFSI DPE/DIPE shifted to 748 and 750 cm^−1^, respectively. The observed shifts indicate that LiFSI in DME underwent significant dissociation, resulting in a highly solvent-rich coordination structure around Li ions. In contrast, the smaller shifts in 2.5 M LiFSI DPE and 2.5 M LiFSI DPE/DIPE imply weaker interactions between Li^+^ and solvent molecules, with stronger FSI^–^ coordination with Li^+^, particularly in 2.5 M LiFSI DPE/DIPE [1, 4, 42, 43]. Further analysis of the Raman spectra (Fig. S4) revealed that in 2.5 M LiFSI DME, approximately 75.624% of FSI^–^ ions remained unbound (free FSI^–^), while the remaining 24.376% entered the Li^+^ solvent sheath and formed contact ion pairs (CIPs). In 2.5 M LiFSI DPE, the proportions of FSI^–^, ion aggregates (AGGs) and CIPs are about 0.000%, 45.510% and 54.490%, respectively. 2.5 M LiFSI DPE/DIPE, the proportions of FSI^–^, ion aggregates (AGGs) and CIPs are ~ 0.000%, 45.513%, and 54.487%, respectively. This indicates that compared to 2.5 M LiFSI DME, a higher proportion of FSI^–^ ions engage in coordination with Li^+^ in 2.5 M LiFSI DPE and 2.5 M LiFSI DPE/DIPE.

To achieve a more thorough comprehension of the solvent structure in 2.5 M LiFSI DME, 2.5 M LiFSI DPE, and 2.5 M LiFSI DPE/DIPE, we conducted molecular dynamics (MD) simulations. The Snapshot (Figs. 1c, d and S5a) and radial distribution function (RDF, Figs. 1e, f and S5b) were acquired to reveal that Li^+^ coordination environment. The types of distinct solvation structure clusters (coordination number populations) identified in 2.5 M LiFSI DME, 2.5 M LiFSI DPE, and 2.5 M LiFSI DPE/DIPE are 14, 17, and 25, respectively (depicted in Figs. S5c, 1g, h, and Table S2, S3 and S4). The result demonstrated the highest diversity of solvation structure in 2.5 M LiFSI DPE/DIPE. The spatial distribution function (Fig. S6, yellow for Li^+^, red for FSI^–^, black for DME, blue for DPE, and green for DIPE) also revealed that 2.5 M LiFSI DPE/DIPE exhibited the most pronounced disorder among all the three electrolytes, consistent with the diversity of solvation structure in 2.5 M LiFSI DPE/DIPE. It should be noted that the more diversities of solvation structure and disorder will diminish the binding between of Li^+^ and solvents [44] in 2.5 M LiFSI DPE/DIPE, which will contribute to the desolvation process. Also, the RDF of Li^+^ in 2.5 M LiFSI DME indicated that the oxygen atoms coordinating with Li^+^ comprised 2.66 from FSI^–^ and 2.84 from DME in the first solvation shell (Fig. 1i). In 2.5 M LiFSI DPE, the number of oxygen atoms derived from FSI^–^ and DPE was 2.93 and 1.34, respectively. Remarkably, when DIPE was introduced into the DPE-based electrolyte, the number of oxygen atoms combined with Li^+^ from FSI^–^, DPE, and DIPE was 3.22, 0.66, and 0.47, respectively. In the first solvation shell, the total number of oxygen atoms from DPE and DIPE coordinating with Li^+^ in 2.5 M LiFSI DPE/DIPE was 1.13, which was lower than that in 2.5 M LiFSI DME and 2.5 M LiFSI DPE. The schematic solvent shells of 2.5 M LiFSI in DME, 2.5 M LiFSI DPE, and 2.5 M LiFSI DPE/DIPE (V:V = 1:1) are displayed in Figs. S7 and 1j, k. These results indicate that DPE solvents reduced the number of solvent molecules while increasing the ratio of FSI^–^ in the solvation shell of Li^+^. Importantly, the introduction of DIPE into the 2.5 M LiFSI DPE electrolyte led to fewer solvent molecules and more FSI^–^ anions entering the solvent shell of Li^+^, consistent with the findings from NMR and Raman analyses.

**Fig. 2:**
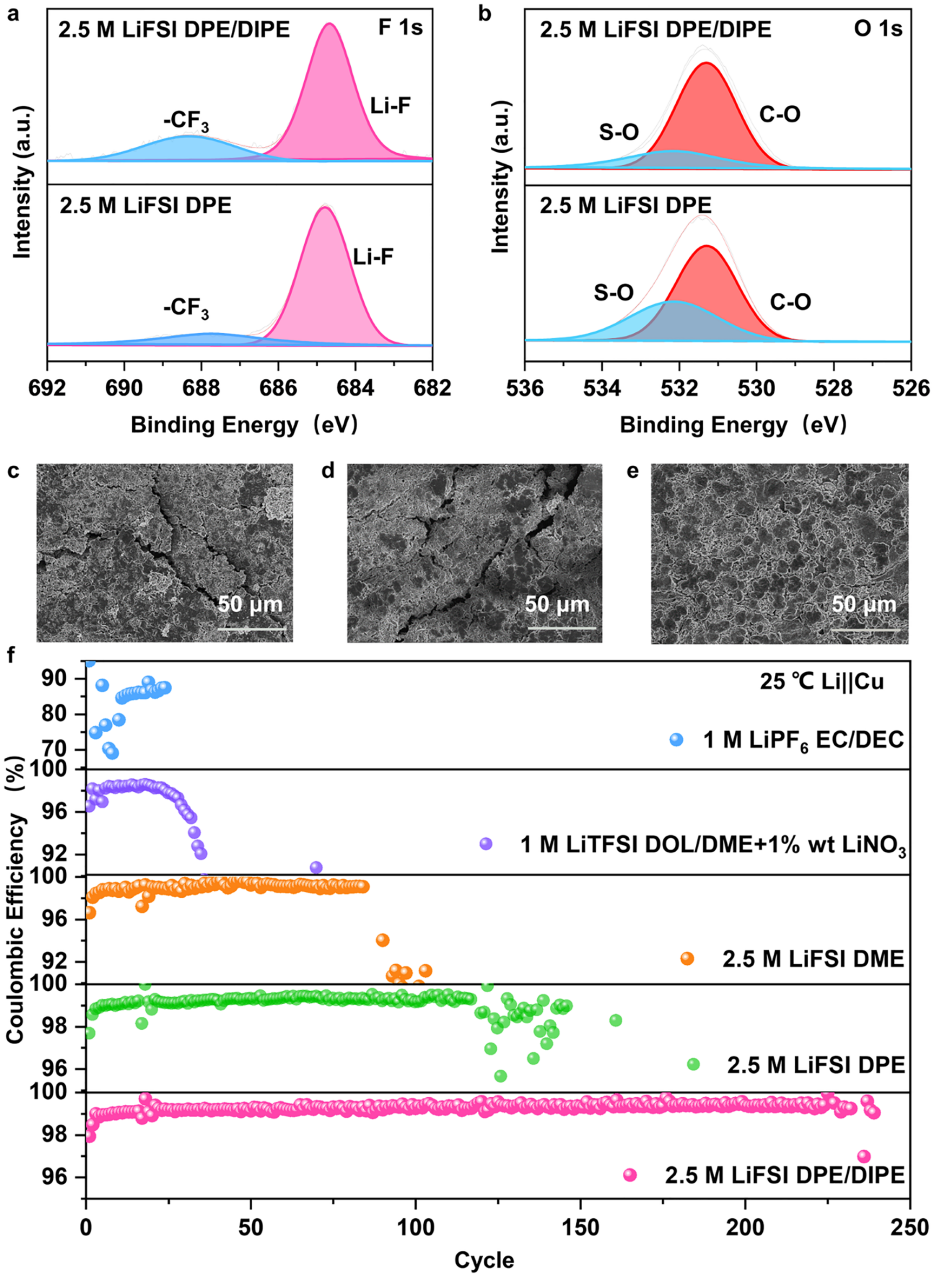
Performance of LMBs at room temperature. **a, b** XPS spectra of the Li surface in Li symmetric cells after three cycles at 1 mA cm^−2^, along with deposition amount of 1 mAh cm^−2^, in 2.5 M LiFSI DPE/DIPE and 2.5 M LiFSI DPE. Li deposition/stripping morphology characterization after 80 cycles in symmetric cells: **c** 2.5 M LiFSI DME, **d** 2.5 M LiFSI DPE, and **e** 2.5 M LiFSI DPE/DIPE under 0.5 mA cm^−2^, maintaining a fixed deposition amount at 0.5 mAh cm^−2^. **f** Li||Cu cell performance using different electrolytes at a current density of 1 mA cm^−2^, with a capacity of 2 mAh cm^−2^

To investigate the impact of different solvation structures on the SEI, we employed X-ray photoelectron spectroscopy (XPS) to analyze the components on the Li surface of the three electrolytes at RT. Notably, the species of the anode after cycling in 2.5 M LiFSI DPE/DIPE, 2.5 M LiFSI DPE (Figs. 2a, b and S8), and 2.5 M LiFSI DME (Fig. S9) were nearly identical at RT, which were similar to the previously reported literature [1]. To assess the influence of the solvent shell structure on the morphology of Li deposition, we assembled Li||Cu half cells and used scanning electron microscopy (SEM) to observe the Li deposit morphologies after plating 6 mAh cm^−2^ of Li on Cu, maintained at a current density of 0.5 mA cm^−2^ at RT. As Fig. S10 illustrates, all three electrolytes provided even and dense surfaces when Li was plated on Cu. We also monitored the morphological evolution of Li||Li symmetric cells over various cycles using SEM. Li deposition was carried out at a capacity of 0.5 mAh cm^−2^ with a current density of 0.5 mA cm^−2^. Li deposition in 2.5 M LiFSI DME and 2.5 M LiFSI DPE both exhibited cracks on the Li surface after 80 cycles (Fig. 2c, d). These cracks increased the specific surface area of the anode and accelerated side reactions between Li and the electrolyte. In contrast, no cracks were observed on the surface of the electrode cycled in 2.5 M LiFSI DPE/DIPE (Fig. 2e). These results demonstrated that Li in the 2.5 M LiFSI DPE/DIPE electrolyte exhibited high reversibility during battery cycling, promoting a more even and stable deposition.

To further understand the 2.5 M LiFSI DPE/DIPE electrolyte, ionic conductivity of various electrolytes was performed (Fig. S11). At 0 and − 20 °C, the conductivity of 2.5 M LiFSI DME electrolyte rapidly decreases to ~ 0.135 and ~ 0.044 mS cm^−1^, respectively. This reduction is probably due to the increase of viscosity in 2.5 M LiFSI DME at lower temperatures. However, the decrease in conductivity for both the 2.5 M LiFSI DPE electrolyte and 2.5 M LiFSI DPE/DIPE electrolyte is not dramatically severe at low temperatures. The ionic conductivities of 2.5 M LiFSI DPE at 0 and − 20 °C are 1.382 and 0.529 mS cm^−1^, respectively. In 2.5 M LiFSI DPE/DIPE, the ionic conductivities at 0 and − 20 °C are 1.162, 0.411 mS cm^−1^, respectively. It should be noted that due to branch chains of DIPE, the ionic conductivity slightly decreases as DIPE was introduced at the given temperatures. However, probably due to the high level of disorder and diversity of solvent structure [44] in 2.5 M LiFSI DPE/DIPE, the decline of ion conductivity was not particularly pronounced as the proportion of DIPE increased**.**

Additionally, to confirm the effectiveness of 2.5 M LiFSI DPE/DIPE in cells, Li||Cu cells were also tested in the three electrolytes and commercial electrolytes, such as 1 M LiPF_6_ EC/DEC and 1 M LiTFSI DOL/DME + 1 wt% LiNO_3_. Each cell was cycled with a current density of 1 mA cm^−2^ and a capacity of 2 mAh cm^−2^ after an activation process (Fig. S12, 0.1 mA cm^−2^, between 0 and 1 V). The cells in 1 M LiPF_6_ EC/DEC and 1 M LiTFSI DOL/DME + 1%wt LiNO_3_ showed poor cyclability, with only 24 and 35 cycles, and average CE values about 84.39% and 97.37% (Fig. 2f). However, half cells in 2.5 M LiFSI DME, 2.5 M LiFSI DPE, and 2.5 M LiFSI DPE/DIPE cycled over 84, 146, and 225 cycles, with average CE values about 99.01%, 99.06%, and 99.28%, respectively.

**Fig. 3:**
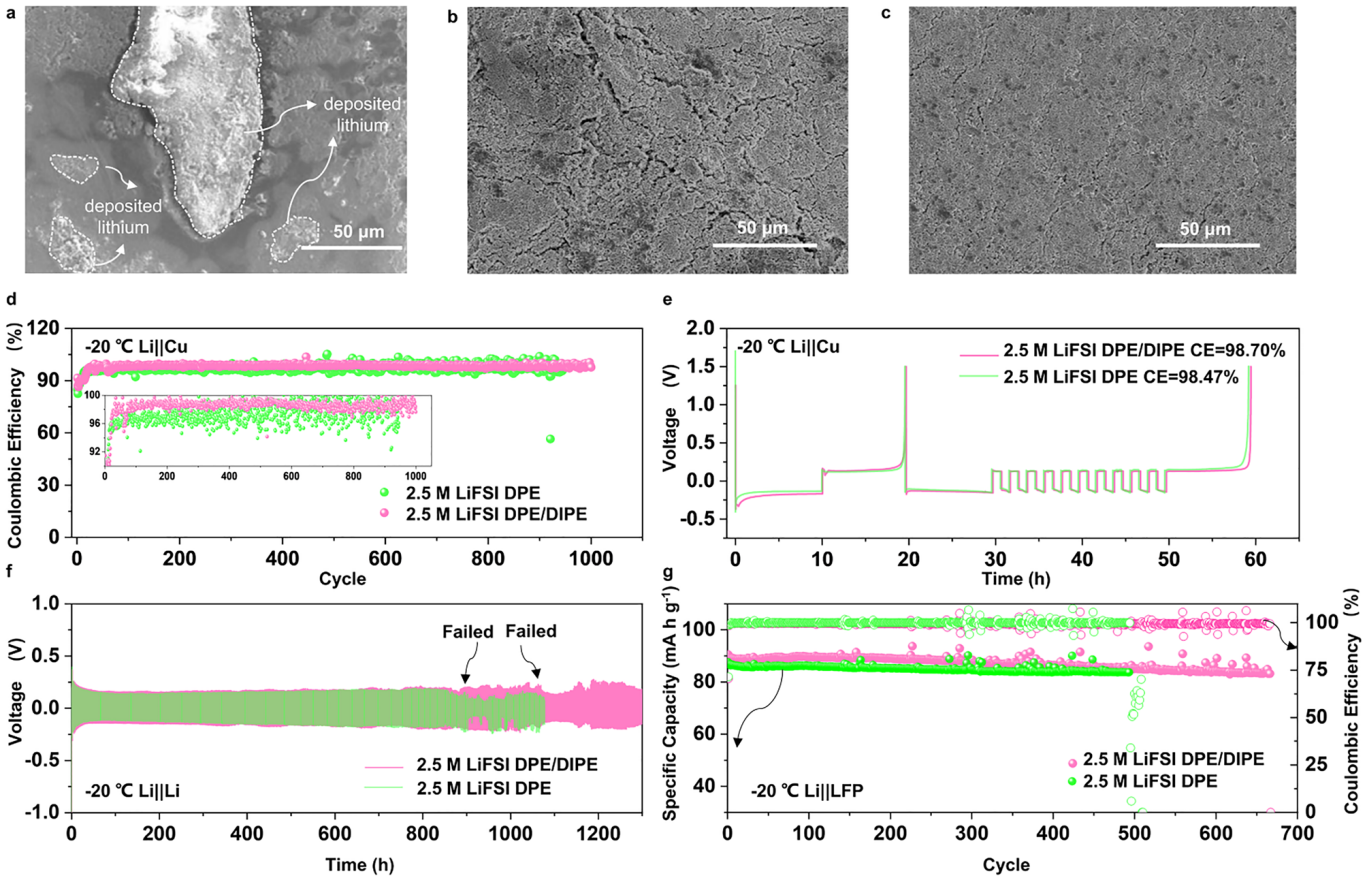
Performance of LMBs at − 20 °C. Morphological characterization of Li deposition/stripping after 40 cycles in symmetric cells: **a** 2.5 M LiFSI DME, **b** 2.5 M LiFSI DPE, and **c** 2.5 M LiFSI DPE at 0.5 mA cm^−2^ and 0.5 mAh  cm^−2^. **d** Cycling performance of Li||Cu cells in 2.5 M LiFSI DPE and 2.5 M LiFSI DPE electrolytes at 0.25 mA cm^−2^ and 0.25 mAh  cm^−2^. **e** Accurate CE test of Li||Cu cells at 0.5 mA cm^−2^ and 0.5 mAh  cm^−2^. **f** Long cycle performance of symmetric cells in 2.5 M LiFSI DPE and 2.5 M LiFSI DPE/DIPE electrolytes at 0.5 mA cm^−2^ and 0.5 mAh  cm^−2^. **g** Full cell performance in 2.5 M LiFSI DPE and 2.5 M LiFSI DPE/DIPE electrolytes at 0.1 C

To further assess the impact of solvated structures on the stability of LMBs, especially at LTs (− 20 °C), the morphology evaluation of the Li surface in symmetric cells after cycling over various cycles was conducted. At the 40th cycle, the deposition of Li in 2.5 M LiFSI DME exhibited significant unevenness surface, as shown in Fig. 3a. Cracks appeared on the anode surface in 2.5 M LiFSI DPE (Fig. 3b). In contrast, the plating on the Li anode in 2.5 M LiFSI DPE/DIPE still maintained a uniform morphology after 40 cycles (Fig. 3c). When the cycle number was increased to 80 (Fig. S13), uneven deposition persisted in 2.5 M LiFSI DME, and the cracks became more severe in 2.5 M LiFSI DPE. However, there were fewer cracks in 2.5 M LiFSI DPE/DIPE. We assessed the CE of Li||Cu cells using the three ether-based electrolytes at LTs. The cell using 2.5 M LiFSI DME showed extremely poor cyclability, as displayed in Fig. S14, quickly failing at − 20 °C. The cell with 2.5 M LiFSI DPE exhibited an unstable performance with obvious fluctuating CE values but still managed over 840 cycles (Fig. 3d and its embedded figure) with an average CE of ~ 97.20% (after an activation process, Fig. S15). In contrast, the cell with 2.5 M LiFSI DPE/DIPE demonstrated a stable performance over 1,000 cycles with an average CE value of about 98.48% (after an activation process, Fig. S15). Additionally, we used an accurate method proposed by Adams [15, 45] to evaluate the average CE of Li||Cu cells using different electrolytes. The 2.5 M LiFSI DPE/DIPE showed a higher CE (98.70%) than 2.5 M LiFSI DPE (98.47%) (Fig. 3e), and both were superior to 2.5 M LiFSI CH_3_(CH_2_)_3_O(CH_2_)_3_CH_3_ (92.98%, Fig. S16) under the same conditions. Symmetric cells in 2.5 M LiFSI DPE/DIPE at − 20 °C (Fig. 3f) also demonstrated a longer cycle life (1,040 h) compared to those in 2.5 M LiFSI DPE (850 h). These results collectively indicate that the change in the solvent structure introduced by DIPE ensures the robust cyclability of LMBs at LTs. To validate the practical value of the 2.5 M LiFSI DPE/DIPE electrolyte, Li (50 μm)||LFP (~ 10.5 mg cm^−2^) cells were initially conducted at RT. The full cell in 2.5 M LiFSI DME electrolyte only lasted for 4 cycles (Fig. S17). As depicted in Fig. S18, the full cell using 2.5 M LiFSI DPE/DIPE demonstrated a better cycling performance maintaining 142.1 mAh g^−1^ of capacity after 150 cycles, and possessed the longest lifetime over 250 cycles (at a current density of 0.2 C, 1 C = 170 mA g^−1^ based on the cathode). In contrast, the full cell in 2.5 M LiFSI DPE delivered a lower capacity (108.0 mAh g^−1^) after 150 cycles. In addition, the full cell with 1 M LiPF_6_ EC/DEC also displayed a low capacity and exhibited rapid fading (121.1 mAh g^−1^) at 125th cycle. These results confirm that 2.5 M LiFSI DPE/DIPE provides the cell with more reversible electrochemical performance under RT conditions than 2.5 M LiFSI DPE and EC-based electrolytes. Full cells were also tested at − 20 °C and 0.1 C. The cell in 2.5 M LiFSI DME showed no capacity at − 20 °C (Fig. S19). The cell using 2.5 M LiFSI DPE/DIPE electrolyte lasted over 650 cycles with a capacity of 87.2 mAh g^−1^ at 0.1 C (Fig. 3g). However, the cell in 2.5 M LiFSI DPE also exhibited good performance, but lasting over 495 cycles. This evidence further suggests that the solvent structure optimized by DIPE is beneficial for LMBs at LTs.

To analyze why the cell using 2.5 M LiFSI DPE/DIPE performs well at LT, we characterized the solvent structure using Raman spectra of 2.5 M LiFSI DPE/DIPE at 25, 0, and − 20 °C. As shown in Fig. S20, the S–N–S bending peak of FSI^–^ exhibited almost no shift at any of the three temperatures. This result indicates that a weak interaction between Li^+^ and the solvent still exists in the 2.5 M LiFSI DPE/DIPE electrolyte at LT. Additionally, XPS was performed (Fig. S21) after cycling a Li||Li cell in 2.5 M LiFSI DPE/DIPE and 2.5 M LiFSI DPE at − 20 °C. The main components such as LiF and C–C, C–O species still existed. Furthermore, the more content of –CF_3_ species with strong electron-withdrawing ability in SEI derived from 2.5 M LiFSI DPE/DIPE, could modulate the frontier molecular orbitals of SEI, and enhance the anti-reduction ability of the SEI [46], contributing to a stable cycle performance of cells in 2.5 M LiFSI DPE/DIPE at − 20 °C.

**Fig. 4:**
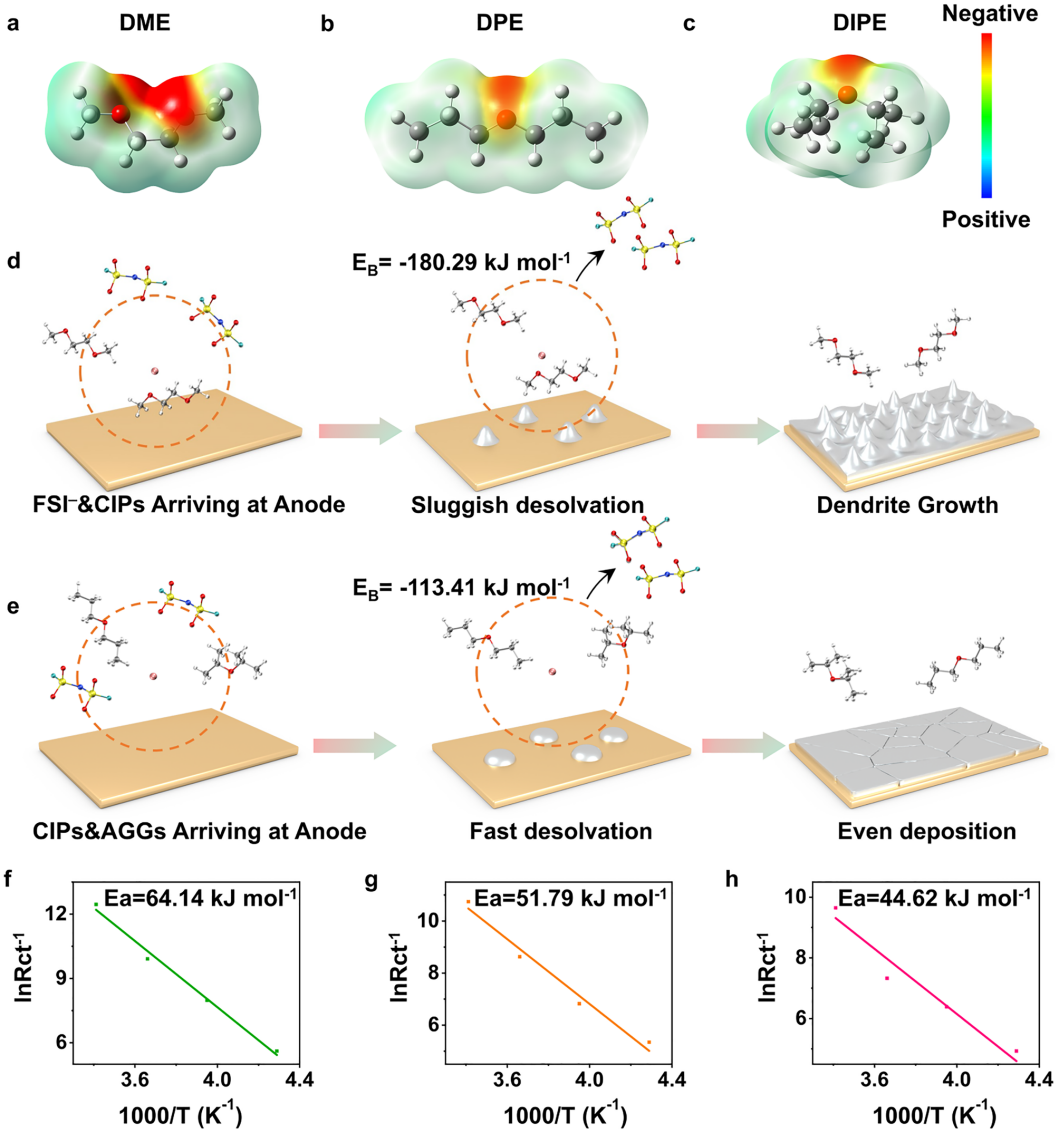
Electrostatic potential of different solvent molecules. **a** DME, **b** DPE, and **c** DIPE. Schematic desolvation process and E_B_ of Li^+^(solvent)_n_ acquired from MD simulations in: **d** 2.5 M LiFSI DME and **e** 2.5 M LiFSI DPE/DIPE. Activation energy calculated from *R*_ct_ in: **f** 2.5 M LiFSI DME, **g** 2.5 M LiFSI DPE, and **h** 2.5 M LiFSI DPE/DIPE

To demonstrate the effect of DIPE on the desolvation process, we assessed the impact of various molecular structures on the binding energies (E_B_) between Li^+^ and the solvents using density functional theory (DFT) calculations. According to previous studies [11], a lower ESP value endows the solvents with nucleophilic capacity, which means that a solvent with a strong negative ESP will strengthen the coordination with Li^+^. As shown in Fig. 4a–c, the charge density around the two O atoms in DME exhibited a more negative value than both DPE and DIPE, resulting in strong coordination between Li^+^ and DME. In contrast, owing to the single oxygen ligand, both DPE and DIPE possess weaker binding abilities than DME. Moreover, when Li^+^ coordinates with the DME molecule, a five-atom ring is formed, which is difficult to separate from each other because of chelation effects [15]. Additionally, as shown in Fig. S22, the ESP_min_ of DIPE was − 1.42 eV, which is more negative than DPE (− 1.36 eV), endowing DIPE with a stronger coordinate ability than DPE. This may result in DIPE competing with DPE to coordinate with Li in 2.5 M LiFSI DPE/DIPE, which changes the solvent structure of Li^+^, inclining toward excluding DPE from the solvent sheath.

The E_B_ of Li^+^ with solvents/anions can also be used to evaluate the desolvation ability of electrolytes. When the solvation shells reach the SEI, the FSI^–^ ions within the shell experience electrostatic repulsion due to the presence of a large negative charge near the anode, causing them to detach rapidly from the solvation sheath [47, 48]. Consequently, the E_B_ between Li^+^ and the anions becomes negligible during desolvation process [49, 50]. Therefore, the desolvation ability of different electrolytes can be assessed based on the E_B_ between Li^+^ and the solvent molecules [1]. The average Li^+^(solvation)_n_ complexes are as follows: Li^+^(DME)_1.42_, Li^+^(DPE)_1.34_, and Li^+^(DPE)_0.66_(DIPE)_0.47_ in the three electrolytes (Fig. 1i). The *E*_B_ for *n* = 1–3 was calculated using DFT, as illustrated in Figs. S23–S25. By fitting the curves of the three types of electrolytes, the *E*_B_ values for Li^+^(DME)_1.42_, Li^+^(DPE)_1.34_, and Li^+^(DPE)_0.66_(DIPE)_0.47_ complexes were determined to be − 180.29, − 136.74, and − 13.4 kJ mol^−1^, respectively. The elevated *E*_B_ of DME resulted in a sluggish desolvation process of Li^+^ at the SEI, leading to poor cycling performance at both RT and LT conditions (Fig. 4d). Furthermore, the reduced E_B_ of DPE and DIPE facilitated the desolvation process within the electrolytes (Fig. 4e), thereby enhancing the stability of LMBs.

To further substantiate the role of DIPE in contributing to a rapid desolvation process, the *R*_ct_ values using different electrolytes was determined over a temperature range from 20 to − 40 °C. The* R*_ct_ of 2.5 M LiFSI DPE/DIPE displayed smallest values at given temperatures, indicating a faster reaction kinetics (as shown in Fig. S26), which promotes a more stable performance of cells. The activation energy (*E*_a_) of Li^+^ diffusion at the SEI film from the *R*_ct_ values were calculated using the Arrhenius equation [51]:

4 $$\frac{1}{{R}_{{\text{ct}}}}=A{\text{exp}}\cdot \left(-\frac{{E}_{a}}{{\text{RT}}}\right)$$

where *A* is the frequency factor, R is the gas constant, and T is the temperature. As depicted in Fig. 4f–h, the *E*_a_ values for 2.5 M LiFSI DME 2.5 M LiFSI DPE, 2.5 M LiFSI DPE/DIPE were 64.14, 51.79, and 44.62 kJ mol^−1^, respectively. The trends in *E*_a_ obtained from *R*_ct_ and *E*_B_ from DFT calculations were consistent, indicating that the desolvation energy barriers were reduced in the mixed electrolyte. This reduction ensures rapid Li^+^/Li charge transfer near the SEI.

**Fig. 5:**
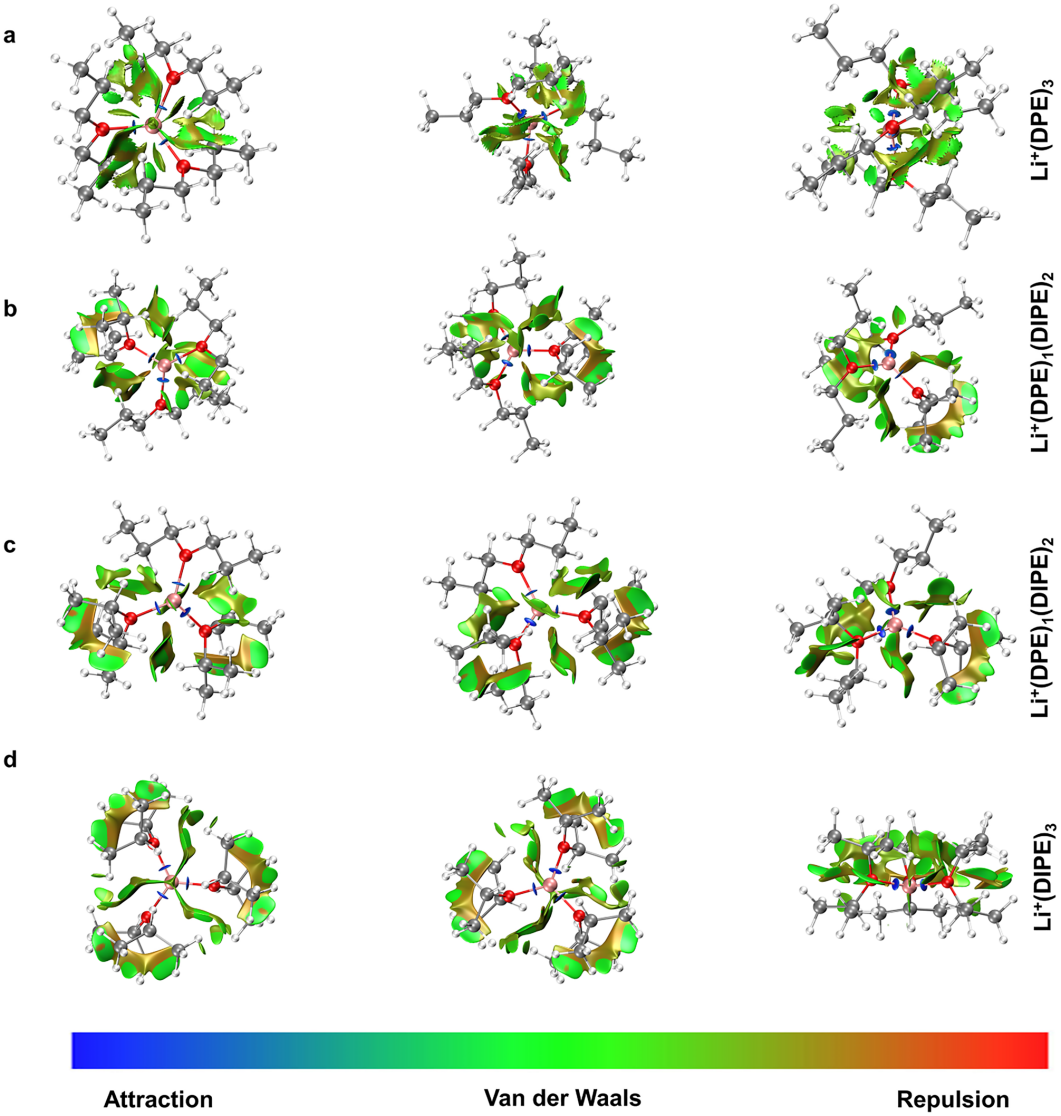
Noncovalent interaction regions obtained from the iso-surface for the **a** Li^+^(DPE)_3_, **b** Li^+^(DPE)_2_(DIPE)_1_, **c** Li^+^(DPE)_1_(DIPE)_2_ and **d** Li^+^ (DIPE)_3_ model

To further investigate why DIPE reduces the desolvation energy barrier of Li^+^, DFT calculations were conducted. Firstly, DIPE competes with DPE to coordinate with Li^+^ in the solvent shell, as confirmed by the ESP analysis mentioned earlier (as shown in Fig. S22). Consequently, the branched chains of DIPE may exert steric effects. When DIPE combines with Li^+^, steric repulsion may occur, hindering or reducing the coordination of Li with other solvent molecules. NCIs were analyzed to confirm the steric repulsion caused by DIPE. Models for Li^+^(DPE)_3_, Li^+^(DPE)_2_(DIPE)_1_, Li^+^(DPE)_1_(DIPE)_2_, and Li^+^(DIPE)_3_ were developed. As shown in Fig. 5a, the green part representing vdW forces dominates the interaction of the DPE molecules in Li^+^(DPE)_3_ with few repulsions (yellow part). However, when the DIPE molecules participated in the Li^+^ solvent shell (Fig. 5b, c), the repulsion (yellow part) increased in Li^+^(DPE)_2_(DIPE)_1_ and Li^+^(DPE)_1_(DIPE)_2_. Besides, when Li^+^ was completely surrounding by DIPE (Fig. 5d), the repulsion will still increase. This evidence indicates that the branched chains in DIPE contribute to increased repulsion among solvent molecules in the shell, thereby enhancing the reduced *E*_B_, fast desolvation of Li^+^, and ultimately leading to the improved performance of the LMBs.

## Conclusions

In this study, we introduced branch-rich DIPE into a DPE-based electrolyte, which significantly improved the electrochemical performance of LMBs at both RT and LT conditions. The DPE/DIPE blend, characterized by its single oxygen ligand DPE/DIPE with a weak binding affinity to Li^+^, was verified using ESP analysis. Furthermore, the increased diversity in the solvation structure and the accompanying disorder within the 2.5 M LiFSI DPE/DIPE system are anticipated to reduce the binding of the Li^+^ with the solvents. Besides, the presence of DIPE with its multiple branched chains led to intermolecular repulsion among solvents within the solvent sheath, a phenomenon attributed to steric effects. This repulsion induced by DIPE played a crucial role in reducing the *E*_B_ between solvent molecules and Li^+^, facilitating a rapid desolvation process. As a result, we achieved even Li plating behavior and stable-long-cycling performance of LMBs at both RT and LT conditions. Notably, this electrolyte demonstrated exceptional performance in Li (50 μm)||LFP cells with a high mass loading of approximately 10 mg cm^−2^, maintaining consistent cycling for over 650 cycles with 87.2 mAh g^−1^ even at − 20 °C. The insights gained from the steric effects explored in this study open up new avenues for designing electrolytes with enhanced stability for LMBs.

## Supplementary Information

Below is the link to the electronic supplementary material.

Supplementary file1 (PDF 2801 KB)
