# Supplementary material for: Branch-Chain-Rich Diisopropyl Ether with Steric Hindrance Facilitates Stable Cycling of Lithium Batteries at − 20 °C
Source: Nanomicro Lett. 2024 May 16;16:197. doi: 10.1007/s40820-024-01419-z (PMC11098989; doi:10.1007/s40820-024-01419-z)
Supplement: Supplementary file 1 — Supplementary file1 (PDF 2801 KB) [file 40820_2024_1419_MOESM1_ESM.pdf]

Supporting Information for

Branch-Chain-Rich Diisopropyl Ether with Steric Hindrance Facilitates Stable Cycling of Lithium Batteries at –20°C

Houzhen Li[1], Yongchao Kang[1], Wangran Wei[1], Chuncheng Yan[1], Xinrui Ma[1], Hao Chen[1], Yuanhua Sang[1], Hong Liu[1,]*, Shuhua Wang[1,]*

[1] State Key Laboratory of Crystal Materials, Shandong University, Jinan 250100, P. R. China

*Corresponding authors. E-mail: hongliu@sdu.edu.cn (Hong Liu); wangshuhua2019@sdu.edu.cn (Shuhua Wang)

Supplementary Figures and Tables

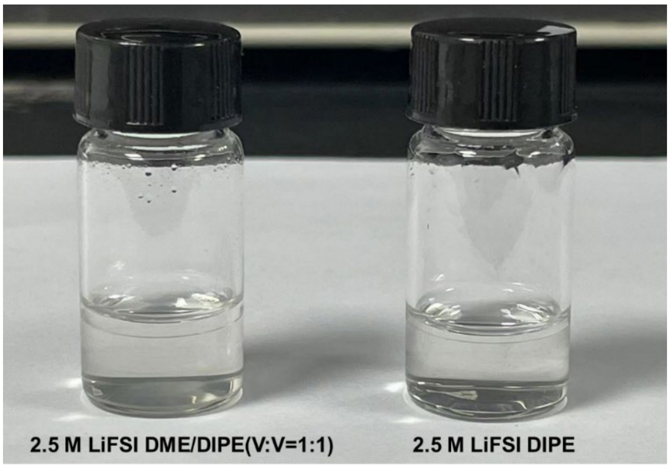

Fig. S1 Stratification phenomenon of 2.5 M LiFSI DME/DIPE and 2.5 M LiFSI DIPE

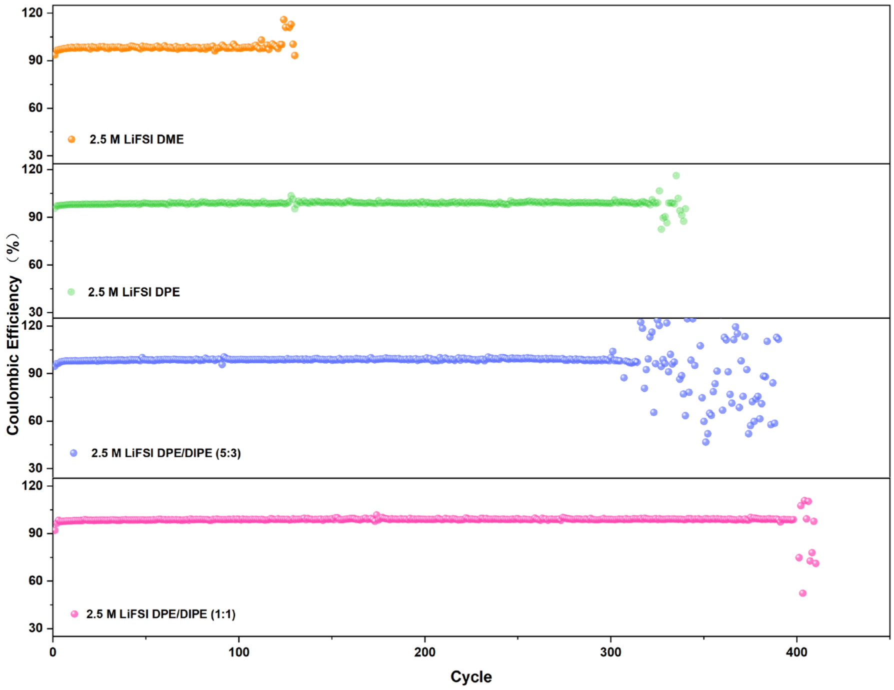

Fig. S2 Cycling performance of Li||Cu cells using different electrolytes at the current density of 1 mA cm^{-2} with the capacity of 1 mA h cm^{-2}

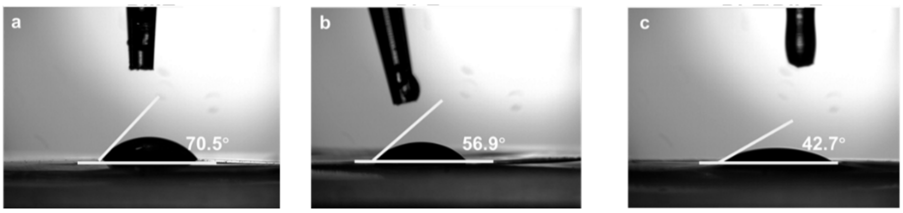

Fig. S3 Contact angle of **a** 2.5 M LiFSI DME, **b** 2.5 M LiFSI DPE and **c** 2.5 M LiFSI DPE/DIPE on Celgard 2325 separators

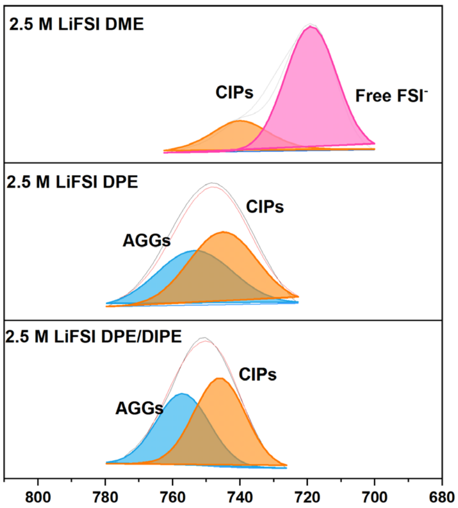

Fig. S4 Deconvolution analyses of S-N-S Raman spectroscopy signal from the electrolytes

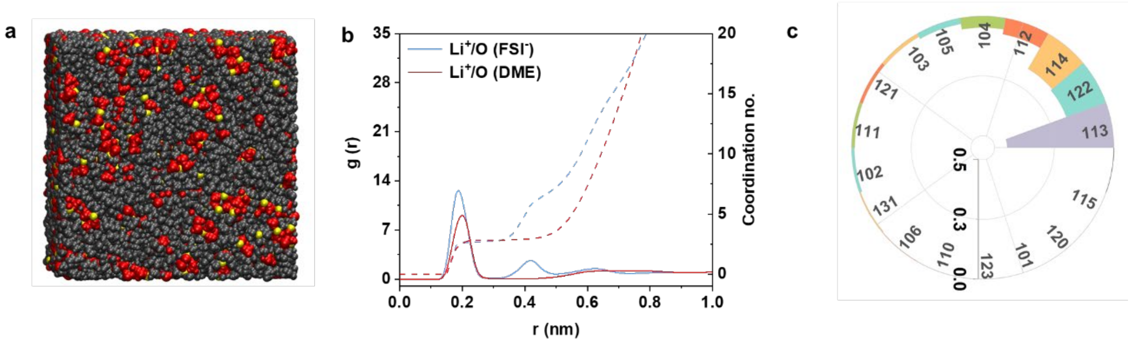

Fig. S5 a Snapshot (yellow parts are Li^+, red parts are FSI^-, black parts are DME) and **b** Li^+ radial distribution function obtained from MD simulations of 2.5 M LiFSI DME and **c** solvent structures (Detailed information can be found in **Table S2**)

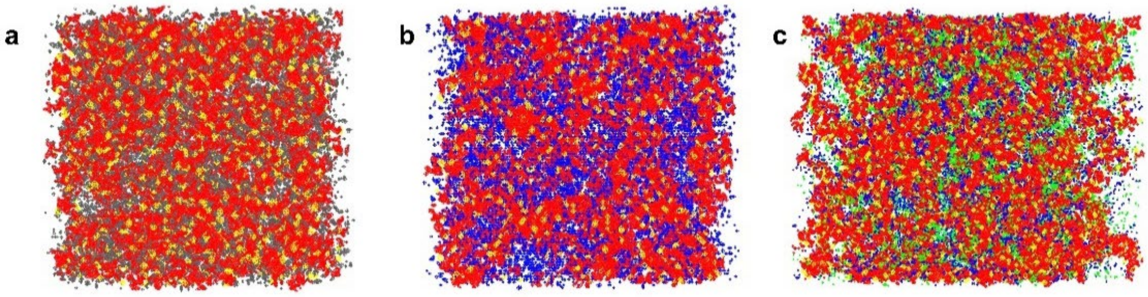

Fig. S6 Spatial Distribution Function obtained from MD simulations of **a** 2.5 M LiFSI DME, **b** 2.5 M LiFSI DPE, and **c** 2.5 M LiFSI DPE/DIPE

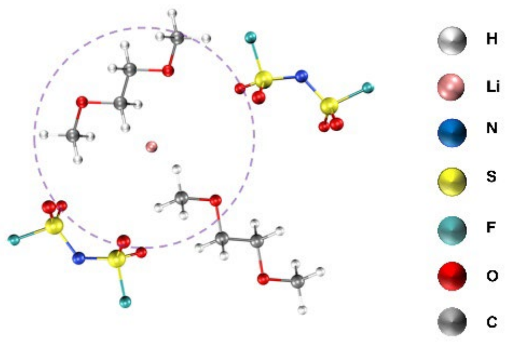

Fig. S7 Schematic diagram of solvent shell structures in 2.5 M LiFSI DME

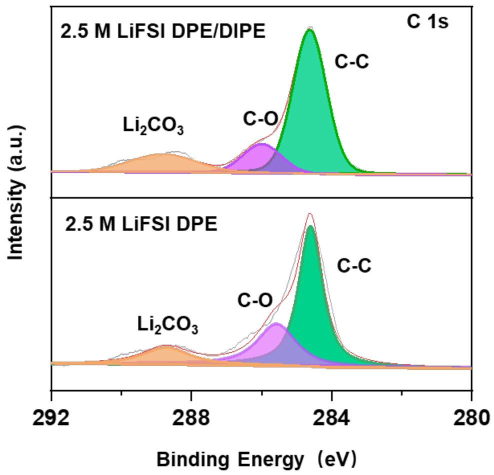

Fig. S8 XPS spectra of the Li surface in Li symmetric cell after 3 cycles with the capacity of 1 mA h cm^{-2} at the current density of 1 mA cm^{-2} in 2.5 M LiFSI DPE/DIPE and 2.5 M LiFSI DPE

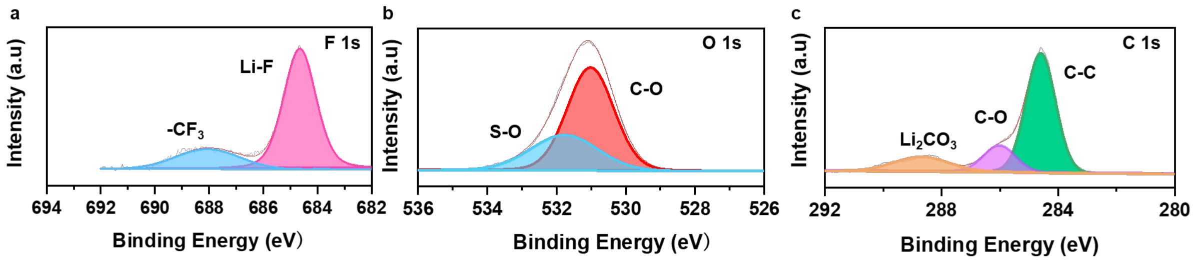

Fig. S9 XPS spectra of the Li surface in Li symmetric cell after 3 cycles with the capacity of 1 mA h cm^{-2} at the current density of 1 mA cm^{-2} in 2.5 M LiFSI DME: **a** F 1s, **b** O 1s, and **c** C 1s

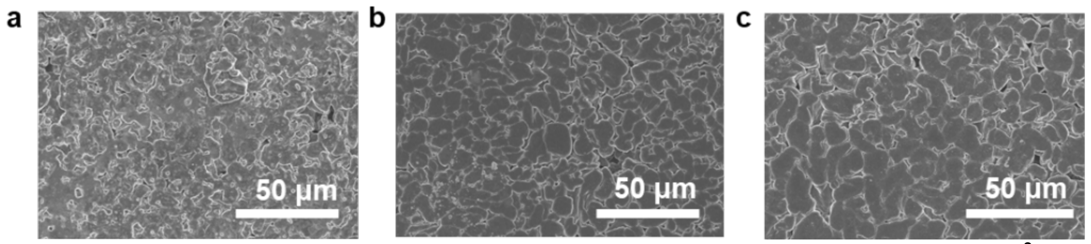

Fig. S10 Morphologies of Li in the three electrolytes after depositing 6 mA h cm^{-2} at 0.5 mA cm^{-2} at RT: **a** 2.5 M LiFSI DME, **b** 2.5 M LiFSI DPE, and **c** 2.5 M LiFSI DPE/DIPE

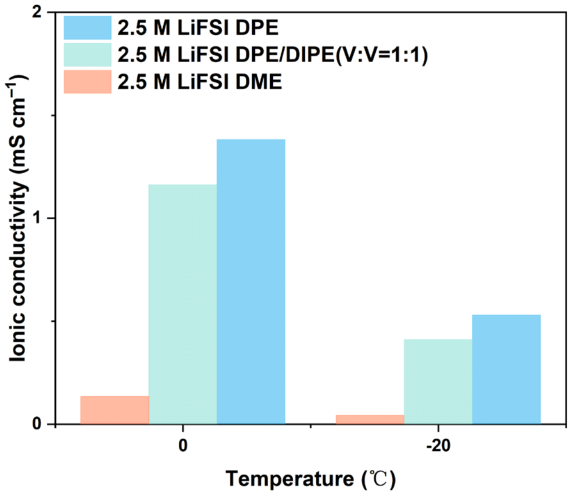

Fig. S11 Ionic conductivity of different electrolytes at various temperatures

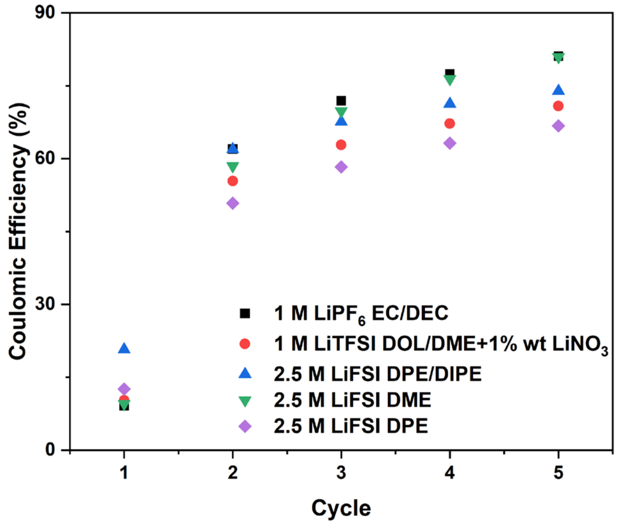

Fig. S12 Li||Cu cell performance using different electrolytes at activation process with a current density of 0.1 mA cm^{-2} between 0-1 V

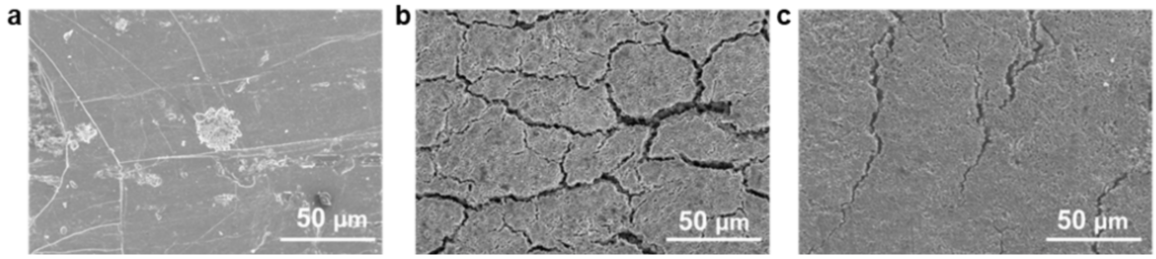

Fig. S13 Li deposition/stripping morphology characterization of symmetric cells after 80 cycles at the current density of 0.5 mA cm^{-2} with the capacity of 0.5 mA h cm^{-2} at –20 °C: **a** 2.5 M LiFSI DME, **b** 2.5 M LiFSI DPE, and **c** 2.5 M LiFSI DPE/DIPE

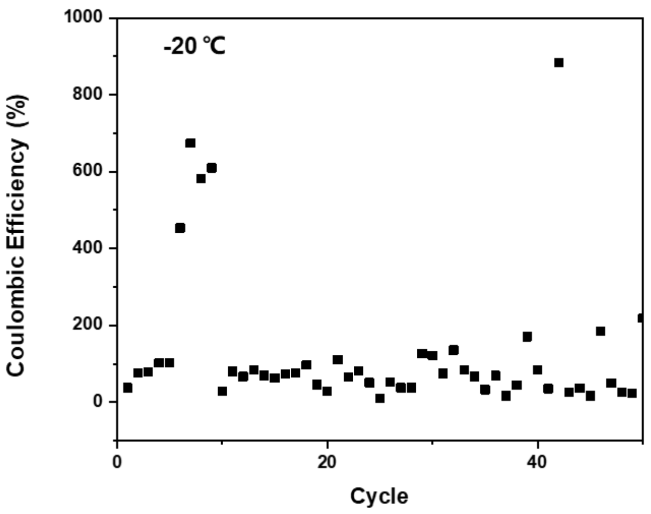

Fig. S14 Cycling performance of Li||Cu cells using DME at the current density of 0.25 mA cm^{-2} with the capacity of 0.25 mA h cm^{-2} at –20°C

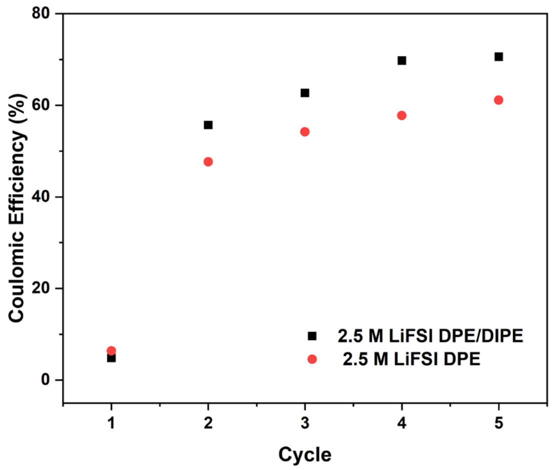

Fig. S15 Li||Cu cell performance using 2.5 M LiFSI DPE and 2.5 M LiFSI DPE/DIPE electrolytes at activation process with a current density of 0.1 mA cm^{-2} between 0-1 V.

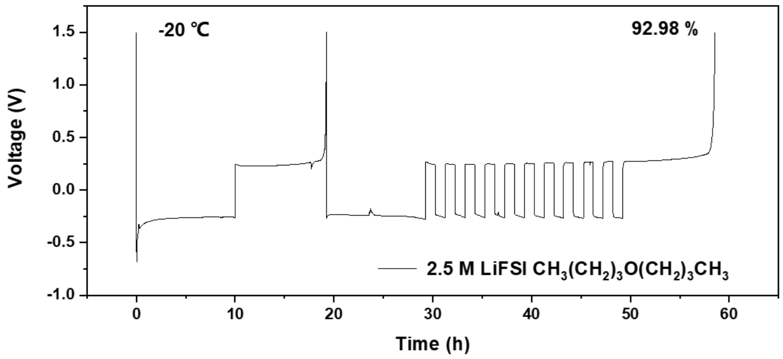

Fig. S16 Accurate CE test of Li||Cu cell in 2.5 M LiFSI $CH_3(CH_2)_3O(CH_2)_3CH_3$ at the current density of 0.5 mA cm^{-2} with the capacity of 0.5 mA h cm^{-2}. (The fluctuations in the curve in Fig. S16 are due to temperature changes caused by placing or removing the battery from the ultra-low temperature refrigerator)

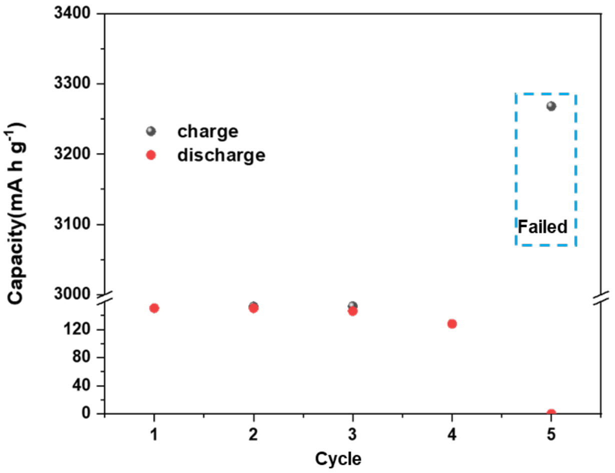

Fig. S17 Cycling performance of Li||LFP cell using 2.5 M LiFSI DME at 0.2 C in RT condition

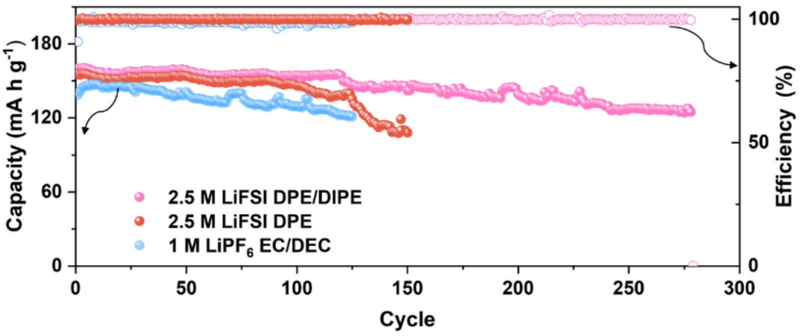

Fig. S18 Cycling performance of Li||LFP cells using 2.5 M LiFSI DPE, 2.5 M LiFSI DPE/DIPE and 1M $LiPF_6$ EC/DEC electrolytes at 0.2 C in RT condition

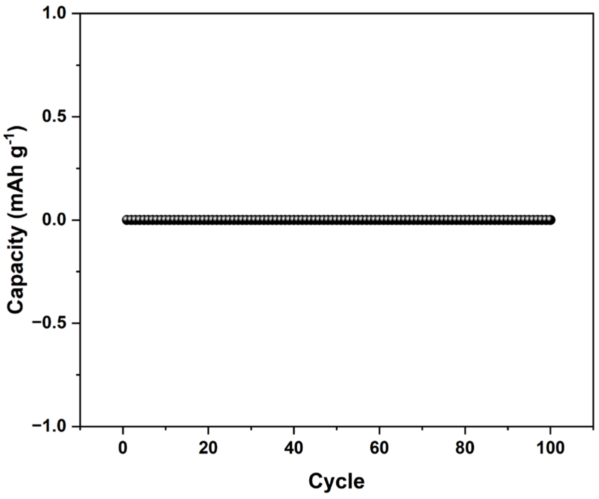

Fig. S19 Cycling performance of Li||LFP cell using 2.5 M LiFSI DME at 0.2 C at –20 °C

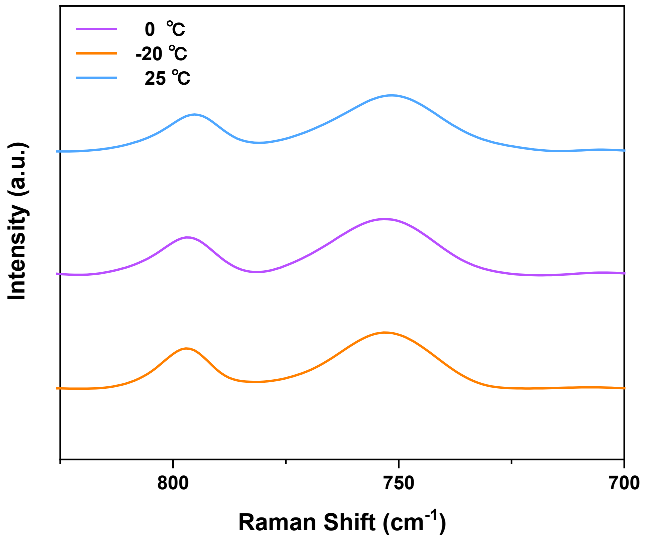

Fig. S20 Raman spectra of 2.5 M LiFSI DPE/DIPE at 25 °C, 0 °C, and –20 °C

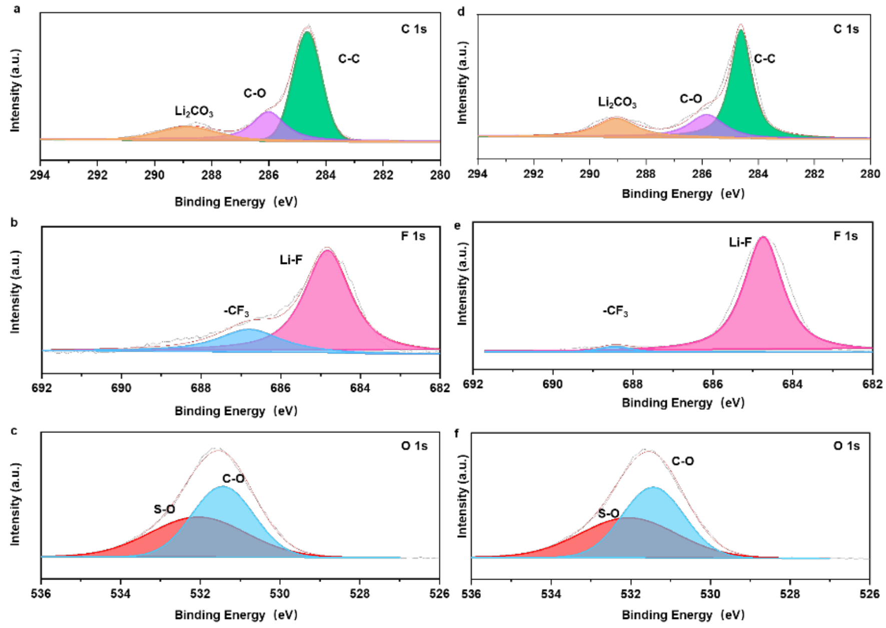

Fig. S21 XPS spectra of the Li surface in Li symmetric cell after 3 cycles with the capacity of 1 mA h cm^{-2} at the current density of 1 mA cm^{-2} in **a-c** 2.5 M LiFSI DPE/DIPE and **d-f** 2.5 M LiFSI DPE

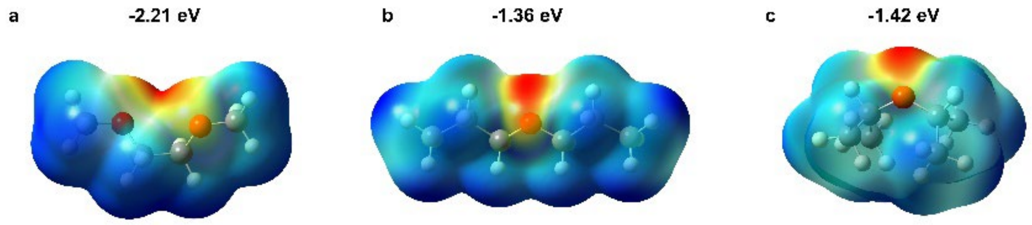

Fig. S22 Electrostatic potential (ESP) of **a** DME, **b** DPE, and **c** DIPE

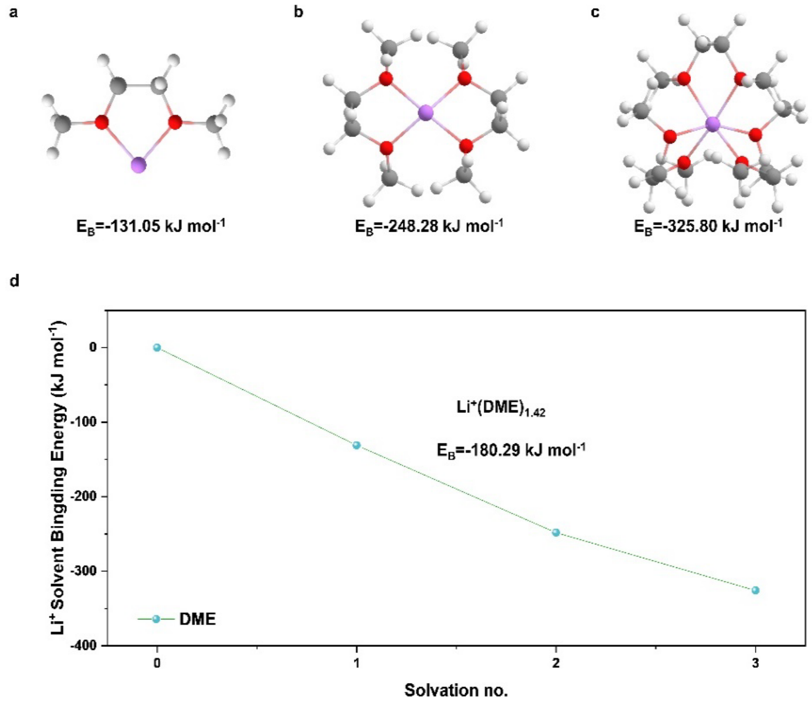

Fig. S23 Optimized structures and calculated binding energies of $Li^+(DME)_n$ solvation complexes. **a** n = 1, **b** n = 2, **c** n = 3. **d** Corresponding binding energies for $Li^+(DME)_n$, n=1, 2, 3. $Li^+(DME)_{1.42}$ values were calculated via linear interpolation

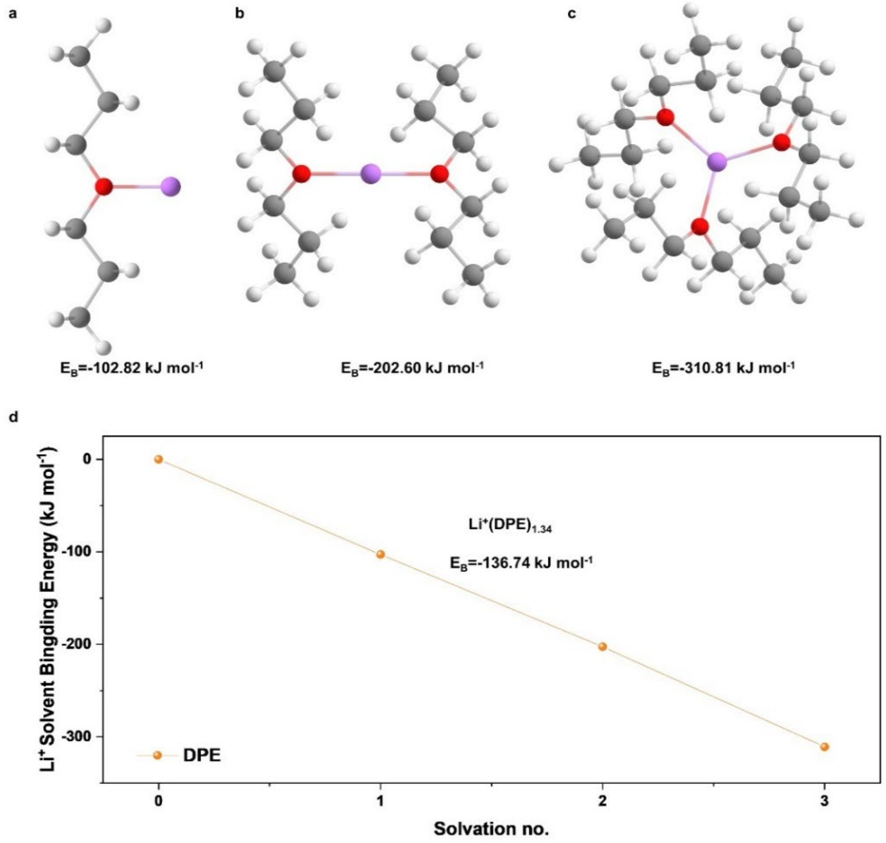

Fig. S24 Optimized structures and calculated binding energies of $Li^+(DPE)_n$ solvation complexes. **a** n = 1, **b** n = 2, **c** n = 3. **d** Corresponding binding energies for $Li^+(DPE)_n$, n=1, 2, 3. $Li^+(DPE)_{1.34}$ values were calculated via linear interpolation

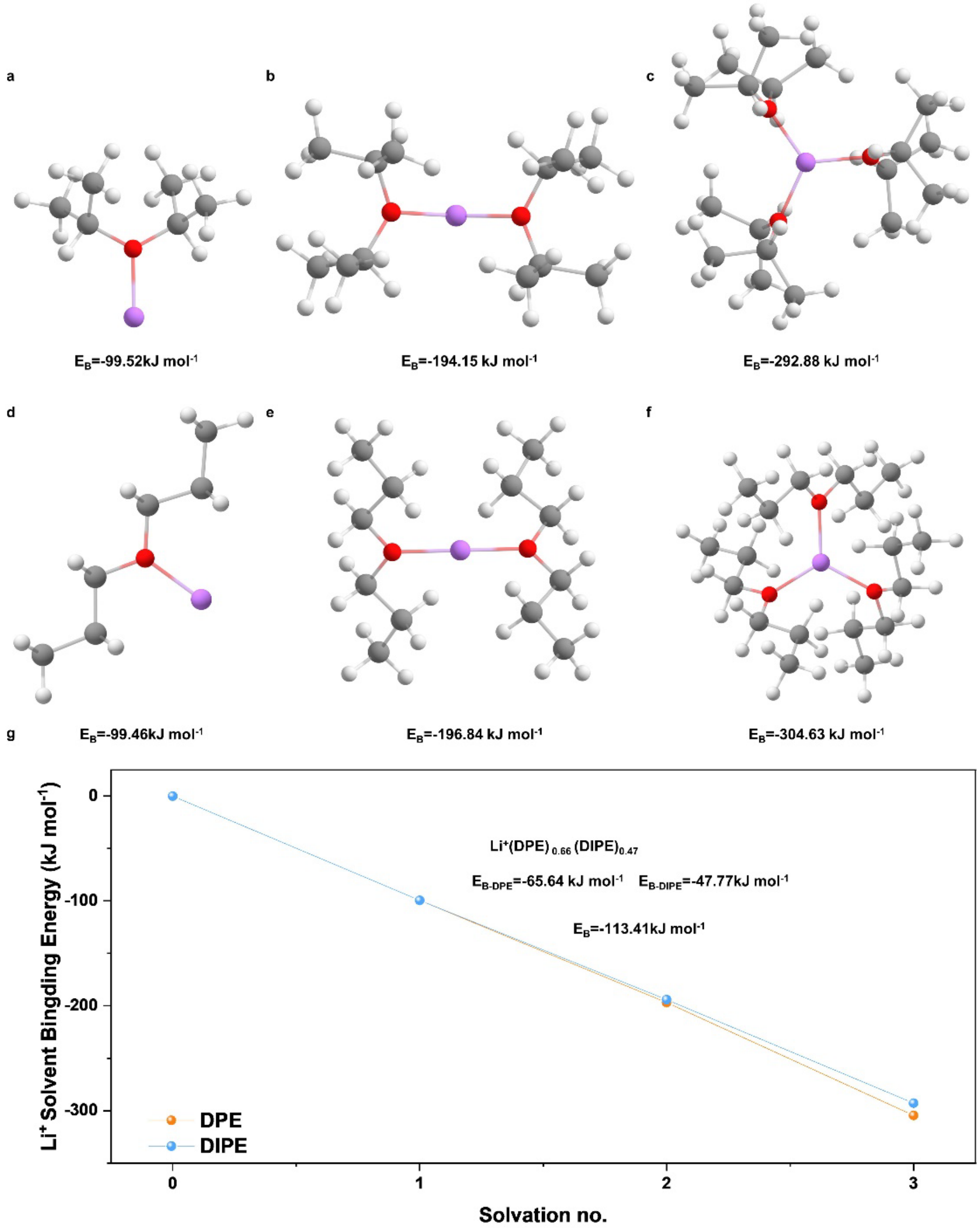

Fig. S25 Optimized structures and calculated binding energies of $Li^+(DPE)_m(DIPE)_n$ solvation complexes. **a** n = 1, **b** n = 2, **c** n = 3, **d** m = 1, **e** m = 2, **f** m = 3. **g** Corresponding binding energies for $Li^+(DPE)_m(DIPE)_n$, m or n=1, 2, 3. $Li^+(DPE)_{0.66}(DIPE)_{0.47}$ values were calculated via linear interpolation

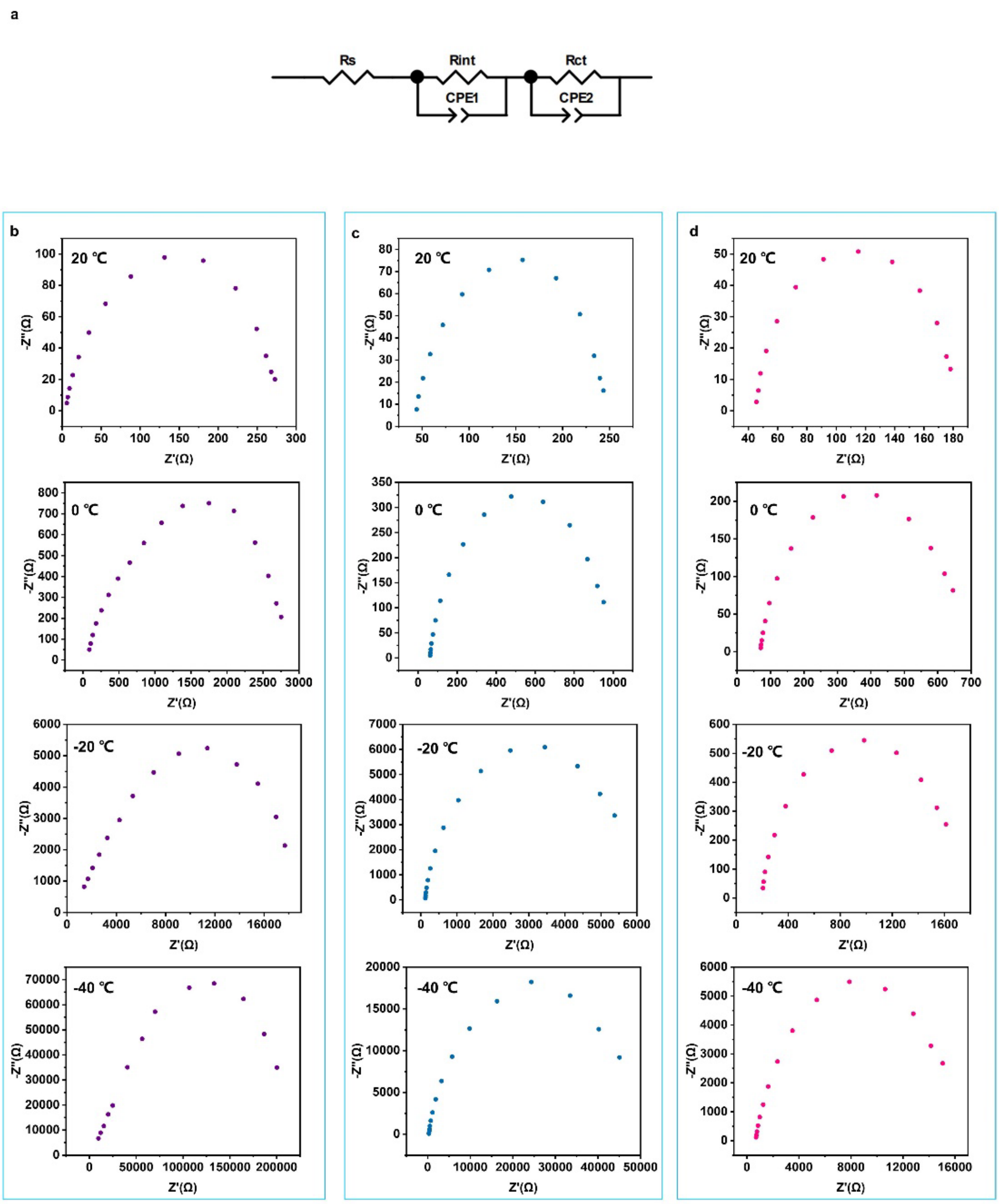

Fig. S26 a Equivalent circuit diagram for Impedance of Li||Li cells. R_{ct} of Li||Li cells in different electrolytes: **b** 2.5 M LiFSI DME, **c** 2.5 M LiFSI DPE and **d** 2.5 M LiFSI DPE/DIPE

Table S1 Solubility, ionic conductivity of different electrolytes and cycle performance of Li||Cu cell in different electrolytes

	2.5 M LiFSI DME	2.5 M LiFSI DPE	2.5 M LiFSI DPE/DIPE (5:3)	2.5 M LiFSI DPE/DIPE (1:1)	2.5 M LiFSI DPE/DIPE (3:5)	2.5 M LiFSI DME/DIPE (1:1)	2.5 M LiFSI DIPE
Solubility	Good	Good	Good	Good	Not completely dissolved	Stratification	Stratification
Ionic conductivity at 25 °C (mS cm^{-1})	7.502	2.077	2.027	2.003	None	None	None
Life of Li||Cu cell at 1 mA cm^{-2} and 1 mA h cm^{-2} (cycles)	111	334	300	398	None	None	None

Table S2 Types of solvation structures and their corresponding proportion in 2.5 M LiFSI DME

Li	DME	FSI^-	Name	Proportion
1	1	3	113	0.37244
1	2	2	122	0.34091
1	2	1	121	0.06322
1	1	4	114	0.05978
1	1	2	112	0.05357
1	0	4	104	0.03686
1	3	0	130	0.0317
1	0	5	105	0.02033
1	1	1	111	0.00879
1	2	0	120	0.00482
1	0	3	103	0.00448
1	0	2	102	0.00241
1	2	3	123	5.16796E-4
1	1	0	110	1.72265E-4

Table S3 Types of solvation structures and their corresponding proportion in 2.5 M LiFSI DPE

Li	DPE	FSI^-	Name	Proportion
1	1	3	113	0.44742
1	2	2	122	0.17701
1	1	4	114	0.1697
1	1	2	112	0.06863
1	0	4	104	0.04795
1	0	5	105	0.02513
1	0	3	103	0.0148
1	2	1	121	0.01373
1	1	1	111	0.01337
1	0	2	102	0.01176
1	3	1	131	0.00535
1	0	6	106	0.00178
1	1	0	110	0.00125
1	2	3	123	0.00125
1	0	1	101	5.34759E-4
1	2	0	120	1.78253E-4
1	1	5	115	1.78253E-4

Table S4 Types of solvation structures and their corresponding proportion in 2.5 M LiFSI DPE/DIPE

Li	DIPE	DPE	FSI^-	Name	Proportion
1	0	1	3	1013	0.26396
1	1	0	3	1103	0.20641
1	0	1	4	1014	0.10593
1	1	0	4	1104	0.06066
1	0	0	4	1004	0.05817
1	0	2	2	1022	0.05646
1	0	0	5	1005	0.04589
1	1	1	2	1112	0.04402
1	1	0	2	1102	0.04402
1	0	1	2	1012	0.04215
1	0	0	3	1003	0.01649
1	0	0	2	1002	0.0098
1	1	1	1	1111	0.00855
1	2	0	2	1202	0.00731
1	0	1	1	1011	0.00684
1	0	2	1	1021	0.00653
1	2	0	1	1201	0.0056
1	1	0	1	1101	0.0056
1	0	0	6	1006	0.00233
1	0	2	3	1023	0.00124
1	0	0	1	1001	0.00109
1	0	3	1	1031	4.66636E-4
1	0	1	5	1015	1.55545E-4
1	0	1	0	1010	1.55545E-4
1	1	0	0	1010	1.55545E-4
